# The association between vitamin D levels and oxidative stress markers in Egyptian Behcet’s disease patients

**DOI:** 10.1186/s13023-022-02416-4

**Published:** 2022-07-15

**Authors:** Heba S. Omar, Fatma Mohamed Taha, Suzanne Fouad, Fatma A. Ibrahim, Aliaa El Gendy, Iman H. Bassyouni, Reem El-Shazly

**Affiliations:** 1 Medical Biochemistry and Molecular Biology Department, Kasr Al Ainy School of Medicine, Cairo University, Kasr Al Ainy St., El Manial, Cairo, 11562 Egypt; 2 Nutrition and Food Science Department, National Research Centre, Dokki, Giza, 12622 Egypt; 3 Biochemistry Department, National Research Centre, Dokki, Giza, 12622 Egypt; 4 Complementary Medicine Department, National Research Centre, Giza, 12622 Egypt; 5 Rheumatology and Rehabilitation Department, Kasr Alainy Hospitals, Cairo University, Kasr Al Ainy st., Cairo, Egypt

**Keywords:** Behcet’s disease, GSH, MDA, Vitamin D

## Abstract

**Background:**

Oxidative stress is postulated to have a major role in the pathophysiology of Bechet’s Disease (BD). Growing evidence suggests that vitamin D has important roles in enhancing the expression of anti-inflammatory cytokines as well as certain antioxidants. However, there is little evidence currently about the antioxidant properties of vitamin D in BD.

**Objective:**

To study the relationship between vitamin D levels and the oxidative stress markers in patients with BD in addition to its association with disease activity and severity.

**Methods:**

Sixty BD patients (45 males, 15 females; mean age: 34.2 ± 9.6 years) were enrolled in this study and compared to a sex and age matched control group. Plasma 25-Hydroxy vitamin D (25-OH-D) was measured using Human (25-OH-D) ELISA assay. Plasma malondialdehyde (MDA), nitric oxide (NO), reduced glutathione (GSH), superoxide dismutase (SOD) activity, catalase (CAT) activity and total antioxidant capacity (TAC) were determined by spectrophotometric methods in both groups. Plasma calcium (Ca) was measured by ELISA assay.

**Results:**

When compared to controls vitamin D, GSH, CAT activity, TAC and Ca were significantly lower in BD patients, while MDA and NO levels were significantly increased in BD patients. Our Results Found that vitamin D was inversely correlated to BD current Activity form (BDCAF), disease severity score, ESR, CRP, MDA and NO, while vitamin D was significantly positively correlated to GSH, SOD, TAC and Ca.

**Conclusion:**

Our study confirms that a lower level of vitamin D is associated with the oxidative stress state in BD patients as detected by MDA and NO elevation as well as decreased GSH, SOD activity, CAT activity and TAC. Hence, Vitamin D fortified foods and beverages or supplementation may improve disease severity and oxidative stress in BD patients.

## Introduction

Behcet’s disease (BD) is a systemic vasculitis with an unknown pathogenesis. It follows a chronic course with inflammatory attacks. The disease is manifested by recurrent oral and genital ulcers, mucocutaneous lesions, vascular, articular, ocular, neurological, cardiac as well as gastrointestinal involvement. BD is of an unknown etiology [1].

Tissue infiltration by active polymorphnuclear cells (PMN) has been detected in BD. Furthermore, their main functions are found to be up regulated in BD i.e. secretion of free radicals (FR), chemoattraction, and phagocytosis [2]**.** The oxidative stress state from enhanced FR secretion may result in an endothelial toxicity and tissue damage in BD [3]**.**

Vitamin D (Vit D) is a fat-soluble vitamin that is essential for normal immune function. A good deal of evidence implicates Vit D in the pathogenesis of several inflammatory diseases including BD [4].

Vitamin D has important roles in enhancing the expression of anti-inflammatory cytokines as well as certain antioxidants [5]. It has been found to regulate the ROS concentrations by its anti-inflammatory functions and mitochondrial secretion of antioxidants through cell signaling pathway [6, 7]**.**

Despite that oxidative stress is involved in the pathophysiology of Behcet’s Disease, limited data related to the effect of vitamin D deficiency on oxidative stress in BD is available. Therefore, we aimed at evaluating the relationship between vitamin D levels and the oxidative stress markers in Patients with Behcet’s Disease (BD) and its association with disease activity and severity.

## Patients and methods

### Study design

This case control study was conducted in Kasr Al Ainy Medical Hospital, The Medical Biochemistry department of Kasr Al Ainy and Excellent center of National Research Centre (NRC) in Cairo in the period between October 2020 and November 2021. This study was approved by the Ethical Committee of Kasr Al Ainy Medical Hospital and was in accordance with the principles of Helsinki Declaration, the protocol of the study was approved by the NRC Ethics committee *(Registration Number is20136)* and all participants provided written informed consent.

### Participants

Ninety five Egyptian subjects participated in the present study. Sixty patients were diagnosed according to the International Criteria for Behcet’s Disease (ICBD) [8]**,** they were recruited from the Rheumatology department of Kasr Al Ainy hospitals, Cairo University & from the Medical Centre of Excellence, National Research Centre, Cairo, Egypt ( 45 males / 15 females; mean age 34.26 ± 6.96 years, mean disease duration 9.55 ± 6.60 years and 35 unrelated healthy controls with similar demographic characteristics (26 males / 9 females, mean age 33.51 ± 6.74 years).

The Detailed medical history and full physical examination were collected by expert rheumatologists. For venous and arterial thrombotic events, Doppler ultrasonography, angiography, MRI, CT, and echocardiography were done. Radiologic evaluations, including, CT scans of the lungs and the abdomen, cranial magnetic resonance imaging (MRI) were performed as clinically indicated. Behcet's Disease current Activity form (BDCAF) was used for assessment of disease activity [8].

The clinical severity score for BD [9] was calculated as the sum of 1 point each for mild symptoms (oral aphthosis, genital ulcers, arthralgia and typical skin lesions such as, erythema nodosum, papulopustular lesions and folliculitis), 2 points each for moderate symptoms (arthritis, anterior uveitis, gastrointestinal involvement and deep vein thrombosis of the legs) and 3 points each for severe disease manifestations (retinal vasculitis, posterior/panuveitis, arterial thrombosis, neuro-Behçet's and bowel perforation). Patients were categorized according to the disease severity score as follows: severe group ≥ 7 points, moderate group, a score between 4 and 6 points and mild group < 4 points.

**Patients Exclusion criteria** were: diabetes; neoplasia; cigarette smoking; other autoimmune diseases; pregnancy; chronic renal failure; liver disease; thyroid disorders; parathyroid disorders; fibromyalgia; antioxidants, vitamin D and calcium supplementation within three months prior to the study.

### Dietary recalls

Collecting detailed basal data about nutritional habits and intake through 24 hour recalls was recorded. Analysis of micronutrients amounts in food intake using World Food Dietary Assessment System (WFDAS) 1995, University of California—USA. Recommended Dietary Allowance (RDA) is the average daily dietary intake level of a nutrient considered sufficient by the Food and Nutrition Board of the Institute of Medicine to meet the requirements of nearly all (97%-98%) healthy individuals.

### Blood samples

Venous blood samples were obtained from patients and controls. Samples were collected into heparinized vacutainers (Becton Dickinson, USA). Heparinized blood samples were used for separation of plasma for the estimation of 25-Hydroxy vitamin D and oxidative stress markers (MDA, GSH, TAC and antioxidant enzymes “SOD and CAT activities”).

#### Biochemical analysis

1. 25-Hydroxy vitamin D Measurement

25-Hydroxy vitamin D (25-OH-D) was measured using Human (25-OH-D) ELISA Assay (epitope diagnostics Co., USA) [10]. Vitamin D ‘deficiency’ was defined as vitamin D levels lower than 20 ng/mL. Vitamin D levels higher than 20 ng/mL and lower than 30 ng/mL were ascribed to vitamin D ‘insufficiency’. Vitamin D ‘sufficiency’ was defined as levels higher than 30 ng/mL [11].

2. Measurement of oxidant/antioxidant parameters

##### Lipid peroxidation

Lipid peroxidation was quantified in the plasma samples by measuring the levels of a secondary product of lipid peroxidation, malondialdehyde (MDA). MDA thiobarbituric acid adducts formed were measured spectrophotometrically at 534 nm [12].

##### Determination of superoxide dismutase (SOD) activity

The method is based on the ability of the enzyme to inhibit the phenazine methosulphate-mediated reduction of nitroblue tetrazolium dye in the plasma. The activity of sample was determined by comparing the increase of absorbance during one minute between sample and blank at 560 nm. Then the percent of inhibition was determined by subtracting the activity of sample from one hundred percent of blank [13].

##### Determination of catalase (CAT) activity

Catalase activity was evaluated by a method of Aeb [14], which is based on decomposition of H_2_O_2_ by catalase. Catalase reacts with a known quantity of H_2_O_2,_ the reaction is stopped after exactly one minute with catalase inhibitor. In the presence of peroxidase (HPR), remaining H_2_O_2_ reacts with 3,5-Dichloro-2-hydroxybenzene sulfonic acid (DHBS) and 4-aminophenazone (AAP) to form a chromophore with a color intensity inversely proportional to the amount of catalase in the original sample.

##### Determination of reduced glutathione (GSH) levels

Reduced glutathione in the blood was determined by the method of Beutler et al. (1963) [15]. GSH was measured by determining the yellow coloured complex formed by the conversion of 5,5′-dithio-bis 2-nitrobenzoic acid (DTNB) to 2-nitro-5-mercaptobenzoic acid in the plasma, which was measured by the spectrophotometer at 405 nm.

##### Determination of total antioxidants capacity (TAC)

Plasma TAC was determined according to the colorimetric method of Koracevic et al. (2001) [16]. The assay measured the capacity of the biological fluids to inhibit the production of thiobarbituric acid reactive substances (TBARS) from sodium benzoate under the influence of the free oxygen radicals derived from Fenton's reaction. This reaction can be measured spectrophotometrically at 532 nm.

##### Nitric oxide (NO) Assay

Nitric Oxide Colorimetric Assay Kit provides convenient measure of total nitrate/nitrite in a simple two-step process. The first step converts nitrate to nitrite utilizing nitrate reductase. The second step uses Griess Reagents to convert nitrite to a deep purple azo compound. The amount of the azo chromophore accurately reflects nitric oxide amount in samples. The resulting azo dye has a bright reddish—purple color which can be measured at 540 nm [17]**.**

3. Determination of Total calcium (Ca)

Calcium was measured by ELISA kit (Glory Science Co., USA).

Laboratory investigations done for all patients and controls included: Complete blood picture (CBC), Erythrocyte sedimentation rate (ESR), C-reactive protein (CRP), serum alkaline phosphatase (ALP), serum Creatinine and serum urea.

### Statistical analysis

The data obtained from the experiments were analyzed using the Statistical Package for the Social Sciences, version 21.0, SPSS Inc, Chicago, Illinois, USA (SPSS). Data were statistically described in terms of mean ± SD, median and interquartile range, or frequencies (number of cases) and percentages when appropriate. Comparison of numerical variables between the study groups was performed using the Student t-test for independent samples in comparing two groups when normally distributed and Mann–Whitney U-test for independent samples when not normally distributed. Comparison of numerical variables between more than two groups was performed using one-way analysis of variance test with post-hoc multiple two-group comparisons in normal data and Kruskal–Wallis test in non-normal data. For comparing categorical data, the χ2 -test was performed. Exact test was used instead when the expected frequency was less than 5. The correlations between the mean of 25(OH) D and other variables were analyzed by the Spearman correlation test. The statistical significant was considered as* P* value < 0.05.

## Results

The main demographic and laboratory parameters of our studied population are presented in Table 1. As shown in the table, we didn’t find any significant differences between BD patients and healthy control subjects regarding their age, gender, ALP, urea, Hb %, and platelets count (*P* > 0.05). On the other hand, there were significant differences as regards Calcium, ESR, CRP, WBCs and creatinine concentrations (*P* < 0.05)**.**

**Table 1:** Comparison of demographic and laboratory parameters in the studied groups

Parameter	Patient group (n = 60)	Control group (n = 35)	*P* value
Age (years)	34.26 ± 6.96	33.51 ± 6.74	ns
Gender (Male/Female)	(45/15)	(26/9)	ns
ESR (mm/H)	25(39.75)*	13(6)*	< 0.001
CRP (mg/L)	9(21.1)*	2.8(2.2)*	< 0.001
Ca (mg/dL)	7.97 ± 1.23	8.74 ± 1.05	0.003
ALP (U/L)	73.21 ± 18.73	68.91 ± 12.45	ns
Urea (mg/dL)	28.36 ± 7.49	25.22 ± 8.75	ns
Creatinine (mg/dL)	0.79 ± 0.14	0.6 ± 0.15	< 0.001
HB (g/dL)	13.32 ± 2.04	13.62 ± 1.32	ns
WBC (× 10^3^/ µl)	8.91 ± 3.13	6.49 ± 1.56	< 0.001
Platelets (× 10^3^/ µl)	287.45 ± 82.23	277 ± 66.64	ns

*ESR* erythrocyte sedimentation rate in the first hour, *CRP* C reactive Protein, *Ca* calcium, *ALP* alkaline Phosphatase, *Hb* hemoglobin, *WBC´s* white blood cells

Data were presented as mean ± SD

^*^: Median (Interquartile Range)

Significant (*P* < 0.05)

ns: non-significant

The mean disease duration was 9.55 ± 6.60 years. At the time of blood sampling, the mean BDCAF activity score was 5.18 ± 3.59 (interquartile range 5), and the total BD severity score was 3.06 ± 2.9 (interquartile range 5). 55 of our patients had history of oral ulceration and 51 of them had a  history of genital ulceration. The clinical characteristics of our patients are  presented in Table 2.

**Table 2:** Behcet’s disease patients' Characteristics

Parameter	BD patients (N = 60)
Disease duration (years)^a^	9.55 ± 6.60
BDCAF^a^	5.18 ± 3.59
Severity Score^a^	3.06 ± 2.9
Clinical involvement ^b^	N0 (%)
Oral ulcers	55 ( 91.66)
Genital ulcers	51 (85)
Pseudofolliculitis/Erythema nodosum	13 (21.66)
Articular involvement	8(13.33)
Ocular involvement	38(63.33)
Neurological involvement	20(33.33)
Vascular involvement	35(58.33)
Current Medications^b^	N0 (%)
CorticoSteroids > 10 mg/day	36 (60)
*Azathioprine*	19(31.66)
*Colchicine*	24(40)
*Cyclosporine*	9(15)
*Cyclophosphamide*	8(13.33)
*Methotrexate*	9(15)
*Infliximab*	15(25)

*BDCAF* Behcet's Disease current Activity form

^a^Continuous variables: mean ± SD

^b^categorical variables: number (%)

Table 3 shows the mean ± SD of micronutrients of the diet of the BD and the control groups; the mean intake of vitamin D and calcium were below the RDA in BD group as compared to controls group (2.01 ± 0.65 µg versus 3.918 ± 0.37 µg) and (519.21 ± 70.61 mg versus 852.73 ± 24.72 mg) respectively, while mean vitamin A and mineral intake of iron, zinc and potassium were appropriate to the RDA requirements in both studied groups.

**Table 3:** Mean ± SD & %RDA of micronutrients food intake analysis by WFDAS of the studied groups

Micronutrient intake	Patient group(n = 60)	Control group(n = 35)	RDA
	Mean ± S D %RDS	%RDS Mean ± S D	
Vitamin. A (µg)	771.54 ± 22.13 96.46%	778.51 ± 21.30 97.12%	800
Vitamin. D (µg)	2.01 ± 0.65 40.20%	75.60% 3.918 ± 0.37	5
Sodium (mg)	320.89 ± 13.85 64.18%	309.78 ± 12.42 61.96%	500
Potassium (mg)	1630.71 ± 24.28 81.54%	1638.89 ± 61.52 81.94%	2000
Calcium (mg)	519.21 ± 70.61 51.924%	852.73 ± 24.72 85.27%	1000
Iron (mg)	11.26 ± 2.99 75.07%	11.30 ± 3.02 75.33%	15
Zinc (mg)	9.76 ± 1.61 81.33%	10.22 ± 1.48 85.17%	12

*WFDAS* World Food Dietary Assessment System, *RDA* Recommended Dietary Allowance by Food and Drug Administration

Plasma levels of 25-Hydroxy vitamin D and oxidative stress markers (MDA, NO, GSH, TAC and antioxidant enzymes “SOD and CAT activities”) are presented in Table 4. Plasma vitamin D levels were significantly lower in BD patients as compared to controls (14.6 ± 5.5 ng/ml) versus (24.5 ± 16.3 ng/ml, *P* = 0.002). Plasma CAT activity, GSH and TAC levels were significantly lower in BD patients (39.86 ± 16.45, 19.33 ± 11.32 and 0.43 ± 0.17,respectivelly) as compared to controls (66.94 ± 33.37, 9.93 ± 2.79 and 1.02 ± 0.44 respectively, *P* < 0.001). BD patients showed a non-significant decrease in plasma level of SOD activity than control. There was a significant increase in plasma MDA and NO levels in BD patients (7.14 ± 1.83, 82.48 ± 29.46, respectively) as compared to control (5.07 ± 2.99, 55.25 ± 26.79, *P* = 0.001, *P* < 0.001, respectively).

**Table 4:** Comparison of 25-Hydroxy vitamin D and oxidant/antioxidant parameters in studied groups

parameter	Patient group (n = 60)	Control group(n = 35)	*P* value
25-OH-D (ng/ml)	14.6 ± 5.5 ( 6–37)#	24.5 ± 16.3 (10–65)#	0.002
MDA(nmol/ml)	7.14 ± 1.83	5.07 ± 2.99	0.001
NO (µmol/L)	82.48 ± 29.46	55.25 ± 26.79	< 0.001
SOD activity(U/ml)	130.84 ± 23.12	140.34 ± 34.88	0.068
CAT activity(U/L)	39.86 ± 16.45	66.94 ± 33.37	< 0.001
GSH(mg/dl)	19.33 ± 11.32	9.93 ± 2.79	< 0.001
TAC(mM/L)	0.43 ± 0.17	1.02 ± 0.44	< 0.001

*25-OH-D* 25-Hydroxyvitamin D, *MDA* Malonaldahyde, *NO* nitric oxide,SOD:superoxidedismutase, *CAT* catalase, *GSH* reduced glutathione, *TAC* total antioxidant capacity

Data were presented asmean ± SD # (Min–Max)

Significant (*P* < 0.05)

**Fig. 1:**
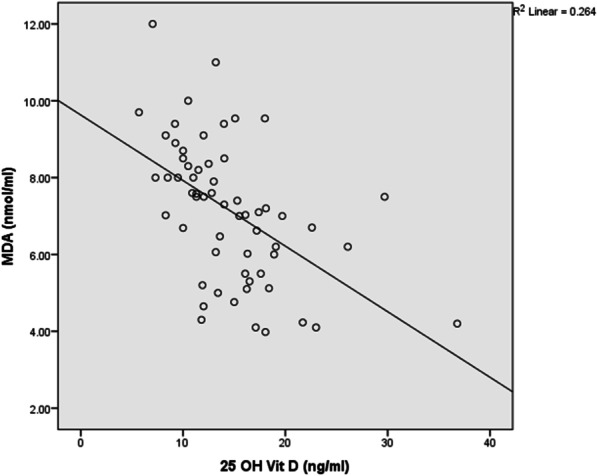
Correlation of Vitamin D with MDA in BD patient

**Fig. 2:**
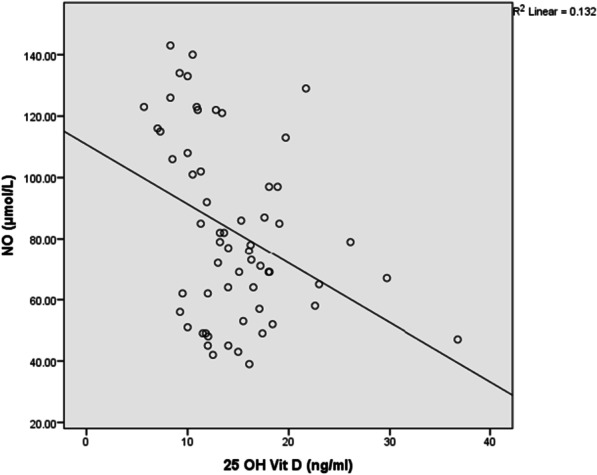
Correlation of Vitamin D with NO in BD patients

**Fig. 3:**
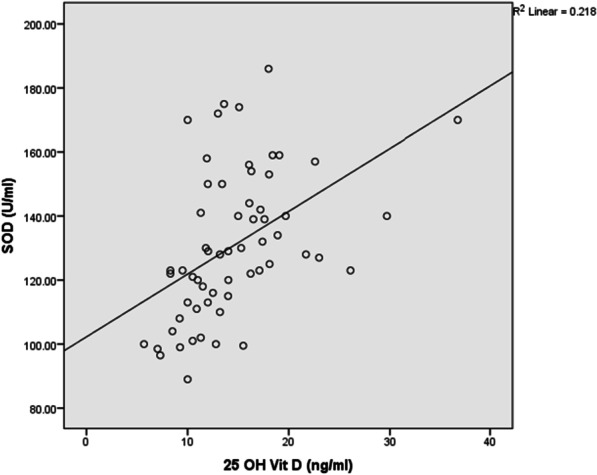
Correlation of Vitamin D with SOD activity in BD patients

**Fig. 4:**
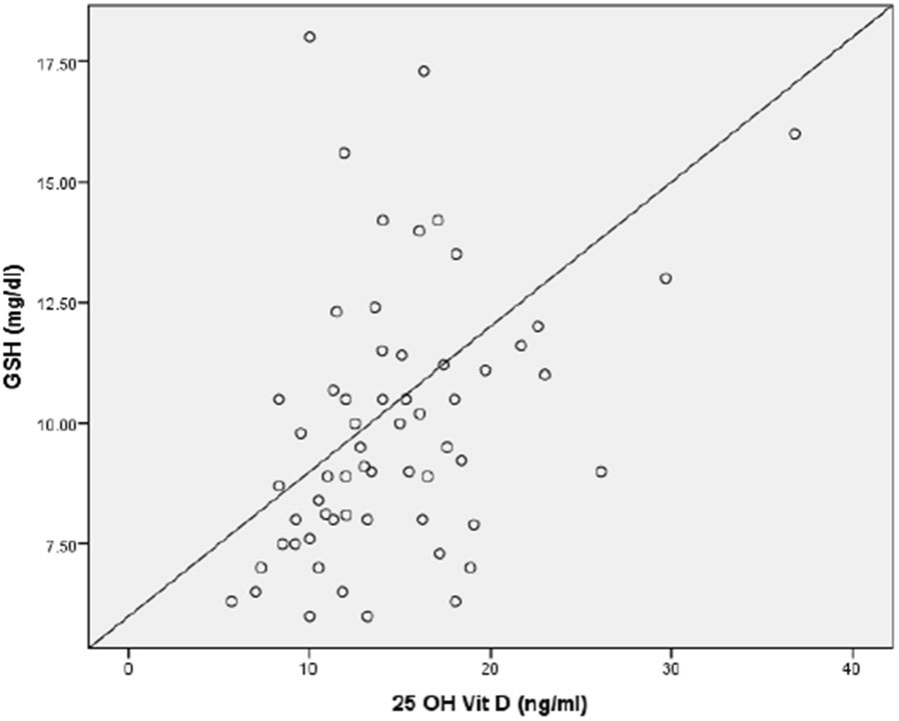
Correlation of Vitamin D with GSH in BD patients

**Fig. 5:**
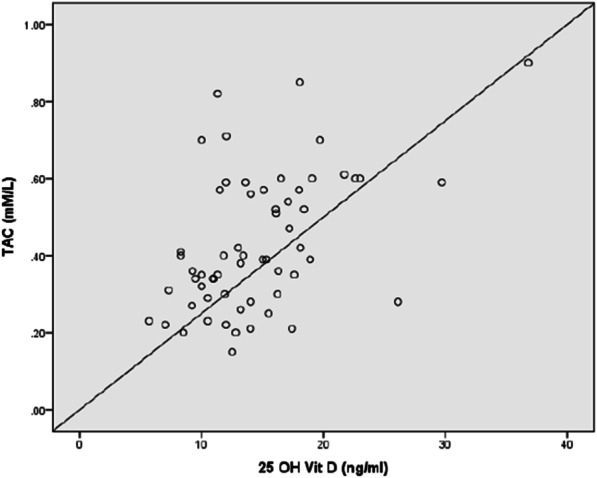
Correlation of Vitamin D with TAC in BD patients

Plasma vitamin D concentrations were negatively correlated with BDCAF (r = − 0.3, *P* = 0.019), severity score (r = − 0.46, *P* < 0.001), ESR (r = − 0.28, *P* = 0.028), CRP(r = − 0.32, *P* < 0.012), MDA (r = − 0.58, *P* < 0.001) (Fig. 1) and NO (r = − 0.35, *P* = 0.005) (Fig. 2).There was a significant positive correlation between plasma vitamin D and Ca (r = 0.45, *P* < 0.001), SOD activity (r = 0.56, *P* < 0.001) (Fig. 3), GSH (r = 0.41, *P* = 0.001) (Fig. 4), and TAC (r = 0.45, *P* < 0.001) (Fig. 5). No significant correlation was seen between Plasma vitamin D levels and age, disease duration, Hb, WBCs, platelet, urea, creatinine, ALP and CAT activity (*P* > 0.05) (Table 5).

**Table 5:** Correlation of 25-Hydroxy vitamin D with demographic, clinical, laboratory and oxidant/antioxidant parameters in Behcet's disease patients

Parameter	Plasma 25-Hydroxy vitamin D (ng/ml)	
	r	*P* value
Age(years)	0.007	0.95
Disease duration (years)	0.068	0.6
BDCAF	− 0.3	0.019
Severity Score	− 0.46	< 0.001
ESR(mm/H)	− 0.28	0.028
CRP(mg/L)	− 0.32	0.012
Ca(mg/dL)	0.45	< 0.001
ALP(U/L)	0.056	0.67
Urea(mg/dL)	0.021	0.87
Creatinine(mg/dL)	− 0.05	0.69
HB(g/dL)	− 0.21	0.1
WBC(× 10^3^/ µl)	0.096	0.46
Platelets(× 10^3^/ µl)	− 0.49	0.25
MDA(nmol/ml)	− 0.58	< 0.001
NO (µmol/L)	− 0.35	0.005
SOD activity(U/ml)	0.56	< 0.001
CAT activity(U/L)	− 0.057	0.66
GSH(mg/dl)	0.41	0.001
TAC(mM/L)	0.45	< 0.001

*BDCAF* Behcet’s Disease current Activity form, *ESR* erythrocyte sedimentation rate in the first hour, *CRP* C reactive Protein, *Ca* calcium, *ALP* alkaline Phosphatase, *Hb* Hemoglobin, *WBC´s* white blood cells, *MDA* Malonaldahyde, *NO* nitric oxide, *SOD* superoxidedismutase, *CAT* catalase, *GSH* reduced glutathione, *TAC* total antioxidant capacity

Significant (*P* < 0.05)

Severity of BD was graded as mild, moderate, and severe.Mild disease severity was found in 40 (66.6%) patients. Moderate disease severity was found in 14 (23.3%) patients. Severe disease was found in 6 (10.1%) patients. On comparing vitamin D level and oxidant/ antioxidant stress markers in the three groups with regard to disease severity. There were significant differences in Vitamin D level, MDA, SOD activity and TAC in the 3 groups (*p* = 0.002, 0.006, 0.012 and 0.035, respectively). No statistically significant difference was found in NO, CAT activity and GSH with regard to the disease severity (Table 6).

**Table 6:** Comparison of 25-Hydroxy vitamin D and oxidant/antioxidant parameters in relation to disease severity score

Parameter	Mild(N = 40)	Moderate(N = 14)	Severe(N = 6)	*P* value
25-OH-D (ng/ml)	15.87 ± 5.1	12.95 ± 6.12	9.87 ± 2.77	0.002
MDA(nmol/ml)	6.63 ± 1.67	7.78 ± 1.62	8.98 ± 1.88	0.006
NO (µmol/L)	76.27 ± 26.16	90 ± 29.62	106.33 ± 38.41	0.086
SOD activity(U/ml)	136 ± 19	121.35 ± 27.39	118.16 ± 29.66	0.012
CAT activity (U/L)	39.98 ± 16.37	41.8 ± 19.21	34.5 ± 9.98	0.7
GSH (mg/dl)	10.37 ± 2.74	9.25 ± 2.97	8.6 ± 2.27	0.1
TAC (mM/L)	0.46 ± 0.17	0.36 ± 0.15	0.31 ± 0.15	0.035

*25-OH-D* 25-Hydroxyvitamin D, *MDA* Malonaldahyde, *NO* nitric oxide, *SOD* superoxidedismutase, *CAT *catalase, *GSH* reduced glutathione, *TAC* total antioxidant capacity

Data were presented as Mean ± SD

Significant (*P* < 0.05)

## Discussion

Behçet’s disease (BD) is a relatively uncommon systemic vasculitis characterized by oral and genital ulcers, ocular and skin lesions as well as other systemic manifestations, its prevalence in Egypt in a multicenter nationwide study on 1526 adult patients is 3.6/100,000 [18]**.**

This study revealed significant decrease in vitamin D level between BD patients and healthy control subjects. Our results agree with other studies reporting vitamin D deficiency during BD [19]. Moreover, serum vitamin D levels were found to be significantly in reverse associated with BDCAF and the severity score of BD. Our findings are in agreement with those previously observed by other researchers [19, 20].

We also found a significant decrease of vitamin D during severe stage of BD compared to mild and moderate stages. These results are consistent with the results observed by Zineb et al. [21] who found the incidence of vitamin D insufficiency (57.57%) during active stage of BD matched to inactive stage BD compared to inactive stage (27.27%) and healthy control. Our results also coincide with the results of Adeeb et al. [6] who stated that vitamin D levels tended to be lower among patients with active disease than among patients without active disease.

In this study we found that vitamin D was inversely correlated with CRP & ESR, although no correlation was found with age, these results are in accordance with previous studies [22, 23].

We also examined the oxidant / antioxidant markers in BD, there was a significant increase in plasma MDA and NO levels in BD patients, while plasma CAT activity, GSH and TAC levels were significantly lower in BD patients as compared to controls. Our findings are in agreement with several previous results [24–26]. The explanation for the lower level of antioxidant enzyme glutathione peroxidase (GSH-Px) in BD patients may be due to the release of superoxide radicals into the circulation,as well as superoxide radicals are produced in excessive amounts by neutrophils and ⁄or increase level of MDA in BD patients, as GSH-Px may become deactivated during oxidative stress, [27] and is blocked by MDA [28].

In the present study there were significant differences in MDA, SOD activity and TAC during severe stage of BD compared to mild and moderate stages while there was no statistically significant difference found in NO, CAT activity and GSH with regard to the disease severity. Pronai and Arimori [29] reported that the superoxide radical binding activity of plasma showed correlation with the activity of the disease. They suggested that the total antioxidant capacity (status) might be decreasing due to the release of superoxides by the PMNLs.

Our results are in agreement with Kiraz et al. [30] who found that serum NO levels were significantly higher in active BD patients than in inactive patients and controls, furthermore they found that their levels were normal in patients with inactive disease compared with controls. Also the pathogenesis of vasculitis in BD is due to NO-associated injury of tissues, especially of the endothelium. On the other hand, SOD may have a protective role against inflammation.

Furthermore, Evereklioglu et al. [31] found a statistically significant difference in serum NO between patients in active and inactive stages of the disease, as well as controls. They hypothesized that increased NO production was involved in the overall inflammatory process of BD and concluded that NO was linked to disease activity. Interestingly in another study, they noted high nitrite levels in patients’ plasma compared to control. During the active stage of the disease, NO production is higher. The monocyte/macrophage system was discovered in an early stage of chronic inflammation and was strongly involved in BD pathogenesis as the main cell source of high NO levels [32].

We found that plasma vitamin D levels were significantly inversely correlated with MDA and NO and was a significantly positively correlated with SOD activity, GSH and TAC while, there was no significant correlation between Plasma vitamin D levels and CAT activity. These results implied that vitamin D deficiency increase the oxidative stress in BD patients. Calcitriol has been shown to improve the ROS elimination pathway by increasing the intracellular pool of reduced GSH, partially through upstream regulation of the glutathione reductase (GR) &glutamate-cysteine ligase (GCL) genes [33]. GCL is a vital enzyme in the production of GSH [34]. Vitamin D and GSH concentrations have been found to have a beneficial relationship [35]. Sardar et al. [36] proposed that vitamin D was an antioxidant as a result of an increase in hepatic GSH levels in rats given cholecalciferol. A clinical investigation found that a vitamin D and calcium supplementation combination was markedly reduced malondialdehyde (MDA) and led to a significant increase in plasma GSH & total antioxidant capacity levels compared to supplementation of either calcium and vitamin D separately [37].

Interestingly, there is evidence in the literature that vitamin D3 plays a major antioxidant role in mature erythrocytes. These findings not only confirm that cholecalciferol has an antioxidant effect [38], but also show that 1,25-dihydroxycholecalciferol may act as a direct antioxidant of membranes by stabilizing and protecting membranes from lipid peroxidation via interactions with their hydrophobic parts [39]**.** Vitamin D3 was found to have an antioxidant impact greater than vitamin E, β -estradiol and melatonin in an in vitro study [7].

In conclusion our study is in agreement with previous studies that reported a decrease in circulating levels of vitamin D in several inflammatory diseases including systemic lupus erythematosus and familial Mediterranean fever [40, 41]. Furthermore, our findings demonstrate that the presence of lower levels of vitamin D is significantly correlated with the existence of an oxidative stress state in BD as shown by the increase of MDA, NO and the diminution of GSH, SOD activity, CAT activity and TAC. Foods rich or fortified with vitamin D, such as eggs, mushrooms, salmon, mackerel and fortified foods or beverages (eg, fortified breakfast cereals or fortified milk and juices) or supplementation can help in management BD related symptoms. It is a must to adjust intake of food enriched and/or fortified with vitamin D or to take a daily vitamin D supplement, that may improve oxidative stress and disease severity in BD.

Finally, there were some limitations to our study. First, despite describing the association between vitamin D deficiency and the antioxidant status, disease activity and severity in our patients, a causal relationship needs to be further investigated. Second, although we evaluated 25(OH) D levels in BD patients, we did not consider seasonal measurement, body mass index, physical activity and the effect of medical therapy. It would be interesting to investigate how the 25(OH) D level is dynamic in relation to the above mentioned factors as a complementary approach in a future study.

## Data Availability

The datasets generated during and/or analysed during the current study are available from the corresponding author on reasonable request.
